# High-risk blastemal Wilms tumor can be modeled by 3D spheroid cultures in vitro

**DOI:** 10.1038/s41388-019-1027-8

**Published:** 2019-09-27

**Authors:** Jenny Wegert, Lisa Zauter, Silke Appenzeller, Christoph Otto, Sabrina Bausenwein, Christian Vokuhl, Karen Ernestus, Rhoikos Furtwängler, Norbert Graf, Manfred Gessler

**Affiliations:** 1 Theodor-Boveri-Institute/Biocenter, Developmental Biochemistry, University of Wuerzburg, Wuerzburg, Germany; 2 Comprehensive Cancer Center Mainfranken, University of Wuerzburg, Wuerzburg, Germany; 3 Experimental Surgery, Department of General, Visceral, Vascular, and Pediatric Surgery, University Hospital of Wuerzburg, Wuerzburg, Germany; 4 Kiel Pediatric Tumor Registry, Section of Pediatric Pathology, Department of Pathology, University Hospital of Kiel, Kiel, Germany; 5 Institute for Pathology, University of Wuerzburg, Wuerzburg, Germany; 6 Pediatric Oncology and Hematology, Children’s Hospital, Saarland University and Saarland University Medical Centre, Homburg, Germany

**Keywords:** Paediatric cancer, Embryonal neoplasms, Cancer models

## Abstract

In vitro models represent a critical tool in cancer research to study tumor biology and to evaluate new treatment options. Unfortunately, there are no effective preclinical models available that represent Wilms tumor (WT) — the most common pediatric renal tumor. Especially the high-risk blastemal WT subtype is not represented by the few primary cell lines established until now. Here, we describe a new 3D approach for in vitro cultivation of blastemal WT cells, where primary cultures grown in suspension as spheroids could be propagated long-term. Besides blastemal cultures, we could generate spheroids representing epithelial and stromal WT. Spheroid cultures were analyzed by immunohistochemistry in comparison to corresponding tumor sections and were further characterized by RNA sequencing. Histological appearance of spheroids resembled the original tumor and they expressed marker genes characteristic of early renal development and blastemal WT elements. The cultures were amenable to genetic manipulation and they formed xenograft tumors, which resemble the primary human tumor. This collection of WT spheroids that carry different genetic drivers forms a long-sought tool for drug testing and in vitro modeling.

## Introduction

Wilms tumor (WT) or nephroblastoma is the most common pediatric renal tumor that is diagnosed at a median age of 3.5 years [1]. In Europe, patients are treated according to the International Society of Pediatric Oncology (SIOP) protocol, with preoperative chemotherapy in most cases, followed by surgery and adjusted postoperative chemo- and radiotherapy. Overall survival is at 90%, but strongly dependent on histology and stage [2]. While stromal- or epithelial-type, triphasic, and regressive tumors are classified as intermediate risk, the blastemal type and tumors with diffuse anaplasia represent the high-risk group [3].

WT as an embryonal tumor is thought to arise from renal precursor cells, which explains the histological heterogeneity reminiscent of embryonal kidney development. While blastemal cells are similar to condensed metanephric mesenchyme, the epithelial cells represent early tubular structures. Stromal cells show greater diversity from fibroblast-like appearance to skeletal muscle or even cartilage differentiation. Thus, analysis of WT provides insight into cancer biology as well as into normal kidney development.

The genetics of WT tumorigenesis is complex with numerous oncogenic drivers identified over the years. Starting with the WT suppressor gene *WT1*, often combined with *CTNNB1* or *WTX* alterations [4], the spectrum expanded to include *TP53* mutations in anaplastic WT. Genome sequencing revealed amplification of *MYCN*, mutations affecting the *SIX1/SIX2* homeobox factors or genes involved in miRNA processing and a series of additional, lower frequency driver mutations [5–7].

Despite high survival rates, side effects and long-term sequelae of chemotherapy call for improved therapeutic strategies with reduced toxicity and novel targets, especially in high-risk WT [8, 9]. Unfortunately, there is a lack of effective preclinical models for functional analysis of tumor driver candidates and for testing of new treatment options. Recapitulation in transgenic mouse models proved to be difficult and limited to combined *Wt1* ablation and deregulation of imprinted *Igf2* [10] or rare *Lin28* overexpression [11]. Xenografts of human tumors have been reported as in vivo models capable of replicating triphasic histology [12–14], but are laborious and expensive. Thus, an in vitro cell culture system is highly needed.

Only few WT cell lines are available, mostly from rare anaplastic tumors with *TP53* mutations [15, 16] and few primary stromal cell cultures derived from *WT1*-mutant tumor samples were described [17]. We previously established a collection of 2D primary cell cultures [18], but these are not immortalized. They represent stromal and epithelial parts of WT, but the challenging blastemal subtype could not be propagated. A xenotransplantation study concluded that even short-term cultivation of blastemal tumor cells abolished their subsequent growth as xenografts [14]. Thus, blastemal cells likely need special factors and interactions to retain their phenotype.

Growth of tumor cells as spheroids or organoids may overcome these limitations as they intrinsically provide more physiological 3D interactions. Indeed, for a number of tumor entities there are organoid protocols available to generate cultures that more closely resemble the original tumor [19]. Most of these are directed at epithelia-derived carcinomas and they may need further adaptation for embryonal (blastemal) tumors.

In the course of establishing 2D WT primary cultures we noticed a subpopulation of cells to form floating spheroids that can likewise be propagated. We have extended this to tumor samples of several histological subtypes from different patients and could establish conditions for efficient long-term cultivation and even xenotransplantation. These spheroids were further characterized by marker analysis and expression profiling and they proved to be amenable to genetic modification.

## Results

### Generation of primary 3D spheroid cultures

Cultivation of minced WT samples in cell culture-grade plastic dishes in a variety of medium compositions has led to the establishment of a series of mostly stromal- or epithelial-like primary cultures [18]. Closer inspection of the supernatant that mainly consisted of cellular debris revealed the presence of small, but expanding clusters of cells in some cultures. This prompted us to specifically cultivate these nonadherent cells using low-attachment plates combined with rotary shaking as used in embryoid body formation [20]. Supplementation of media with ROCK inhibitor was required to prevent anoikis in suspension cell cultures. Five long living spheroid 3D cultures could be obtained from different histological WT subtypes, with one being derived from a mouse xenograft (where suffix -X3/-X20 denotes passage number) (Table 1).

**Table 1:** Patient and tumor characteristics

	WT01^a^	WT02	WT03	WT04	WT05
Sex	Female	Male	Male	Female	Male
Age (months)	23	33	42	50	10
Response to preoperative chemotherapy	Good (80% volume reduction)	Good (70% volume reduction)	Good (70% volume reduction)	Progress	Progress
Tumor type	Regressive (vital part: blastema)	Triphasic	Blastemal	Epithelial, with diffuse anaplasia	Stromal
Stage	III	II	I	I	I (V)
Outcome/event free survival (EFS)	>3.5 years EFS	>1 year EFS	>9 months EFS	>4 years EFS	Lung metastasis at 1 year, >3 years EFS
Histology of starting material	Blastemal xenograft	Blastemal with few stromal elements	Blastemal with few stromal elements	Epithelial	Stromal lung metastasis

^a^Reported as WT046 in [7]

**Fig. 1:**
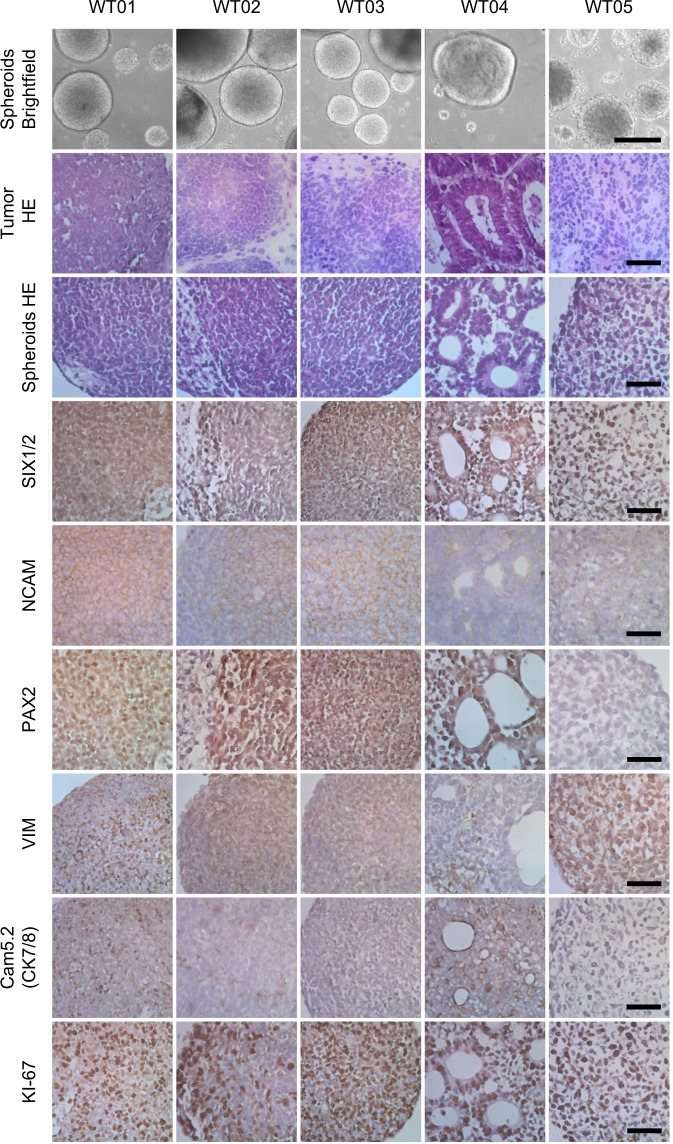
Histology of WT spheroids and corresponding tumor material. Representative spheroids are depicted by low power micrographs (brightfield, row 1). H&E staining showed similar histology of tumor material and corresponding spheroids (rows 2 and 3). IHC for renal progenitor/blastemal markers (SIX1/2, NCAM, and PAX2), the mesenchymal marker vimentin (VIM), the epithelial marker cytokeratin 7/8 (Cam5.2, CK7/8), and the proliferation marker Ki-67 was done on FFPE sections of spheroids. Scale bar: bright field 250 µm, H&E and IHC 25 µm

The culture protocol includes depletion of adherently growing cells from dissociated tumor samples by short-term incubation in cell culture dishes. Nonadherent cells were then cultured in bacterial petri dishes under constant shaking to avoid attachment of cells. Under these conditions cell clusters formed embryoid body-like, solid structures (Fig. 1). Most appeared as homogeneous, unstructured spheres, but in WT04-S internal tubular structures were visible. WT05-S cultures tended to form huge cell clumps upon shaking and were kept on ultralow attachment plates without shaking. For WT03, WT04, and WT05, adherent cells could also be cultured (suffix -A). These resembled the mesenchymal cultures described earlier [18].

Spheroids were passaged by mechanical disruption every 1–2 weeks and they could be cryopreserved with a recovery rate of 60–80% viable cells. Cultures could be kept proliferating for at least 3 months and up to more than 3 years (Table 2).

**Table 2:** IHC analysis and key genetic alterations of spheroid cultures

Spheroids	WT01		WT02		WT03		WT04		WT05	
Life span (months)	3–4		16		7		>11		>36	
Doubling time (weeks)	2		2		1		1		1	
Histology	Blastemal		Blastemal, few stromal elements		Blastemal		Epithelial		stromal	
IHC^a^	Sph	Tu	Sph	Tu	Sph	Tu	Sph	Tu	Sph	Tu
SIX1/2	+++	+++ B	+++ B + S	+++ B − S	+++	+++ B + S	+++	++ E + S	+++	+++ S
NCAM	+++	+++ B	+++ B + S	+++ B − S	+++	++ B − S	+++	+++ E − S	+++	+ S
PAX2	+++	+++ B	+++ B − S	++ B − S	+++	+ B − S	+++	++ E − S	–	− S
VIM	+++	++ B	+++	++ B ++ S	++	++ B ++ S	– ++ S	− E +++ S	+++	++ S
CK7/8	++	++ B	+	++ B − S	–	+ B − S	+++	+++ E − S	–	− S
Ki-67	80%	20%	60%	30%	90%	20%	80%	30%	60%	70%
Key genetic alterations										
Mutations (SNV)	GLI3 Q1437* (NM_000168:exon15:c.C4309T)		MYCN T58M (NM_001293228:exon2:c.C173T), MAX R60Q (NM_002382:exon4:c.G179A)		STK32C V339M (NM_001318878:exon8:c.G1015A)		TP53 G245S (NM_001126112:exon7:c.G733A), CREBBP R1498* (NM_004380: exon27:c.C4492T)		CTNNB1 T41A (NM_001098209:exon3:c.A121G)	
CNV	18p + q gain		Normal		DIS3L2 loss		multiple gains/losses chr.4,6,7,9,10,11,14, 16, 17, 19, 22		1q gain, 7q gain, WT1 loss, 11q23.2 loss	
LOH (regions affected frequently in WT)	11p15-p13		2q11-q37 11p15-p13		11p15		11p15-p13, 11q13-q25, 16q13-q24.3, multiple others		11p15-p13	

^a^Semiquantitative classification of staining: +++ strong, ++ moderate, + weak, − no staining

Restriction to histological compartment if appropriate: *B* blastemal, *E* epithelial, *S* stromal elements

### Characterization of spheroids

As WT displays strong intratumor heterogeneity, spheroids were compared with the corresponding tumor regions they were derived from by histology and genetic characterization. Tumor material was analyzed by whole exome sequencing (WES) for single nucleotide variants (SNVs), copy number variation (CNV), and loss of heterozygosity (LOH) (Supplementary Table S1, Supplementary Fig. S1) and alterations were validated in spheroids by Sanger sequencing, RNAseq, MLPA, or microsatellite PCR, respectively. All spheroid cultures proved to be tumor-derived as they showed the same allele loss patterns, genetic driver mutations, and CNVs as the corresponding tumor material (Table 2). The regressive tumor WT01 carried *GLI3* Q1437*, the triphasic WT02 harbored *MAX* R60Q and *MYCN* T58M mutations, and the blastemal WT03 showed a heterozygous *DIS3L2* deletion and a *STK32C* V339M mutation. The WT04 epithelial tumor with diffuse anaplasia harbored a *CREBBP* R1498* and a *TP53* G245S mutation, while the stromal tumor WT05 had a *CTNNB1* T41A mutation and complete loss of *WT1*.

Histology revealed similar appearances of spheroids and their original tumor material. Three spheroid cultures consisted of mostly blastemal cells (WT01, WT02, and WT03), while one showed predominantly epithelial (WT04) and one immature stromal (WT05) differentiation (Fig. 1). The smaller contribution of stromal cells in WT02 and WT03 was further reduced in spheroids, perhaps due to the initial depletion of adherent cells. The epithelial spheres WT04 still contained interstitial cells, however.

Remarkably, all spheroid cultures expressed the renal precursor markers SIX1/2 and NCAM, irrespective of the histological subtype (Table 2, Fig. 1). SIX1 and NCAM expression is usually not seen in stromal WT elements [14, 21], but was detected in tumor material WT05 (Fig. S2), indicating less differentiated, immature stroma in this tumor and corresponding spheroid cultures. Blastemal and epithelial spheroids were positive for PAX2, a marker of nephron progenitor cells (NPCs), while the stromal cells in WT02 and WT05 were negative. The mesenchymal marker vimentin was detected in all spheroid cultures except WT04. These epithelial spheroids contained very few vimentin positive interstitial cells, but almost all cells were strongly stained with Cam5.2, an antibody detecting cytokeratin 7 and 8, which is typically positive in epithelial regions of WT. Weak staining with Cam5.2 was detected in blastemal spheroid cultures WT01 and WT02, in line with a weak positive staining in blastemal regions of the original tumors (Fig. 1, S2).

### Transcriptome analysis of adherent 2D and spheroid 3D primary WT cultures

RNAseq analysis was performed to elucidate differences between adherent 2D (suffix -A) and 3D spheroid (suffix -S) cultivation of primary WT cells. All spheroid cultures (WT01-X3-S, WT02-S, WT03-S, WT04-S, and WT05-S1) and corresponding adherent cell cultures for three of them (WT03-A, WT04-A, and WT05-A), as well as secondary adherent cells derived from spheroids (WT04-S>A) were analyzed. WT01-X20-S (derived from the 20th xenograft passage) and WT05-S2 (grown in culture for 21 months) were included to assess long-term in vivo or in vitro changes. In addition, early passages of two mesenchymal/stromal adherent WT cultures (WT06-A and WT07-A) were analyzed. They had been established earlier with a slightly different protocol lacking ROCK inhibitor [18] and they did not form spheroids. WT06 and WT07 harbored mutations typical for stromal WT (*WT1* Q252*(NM_024424.3:exon2:c.C757T)/*CTNNB1* S45F (NM_001098209:exon3:c.C134T) and *WT1* R458* (NM_024424.3:exon9:c.C1372T)/*CTNNB1* W383G (NM_001098209:exon8:c.T1147G)) as shown by targeted sequencing and in RNAseq reads.

**Fig. 2:**
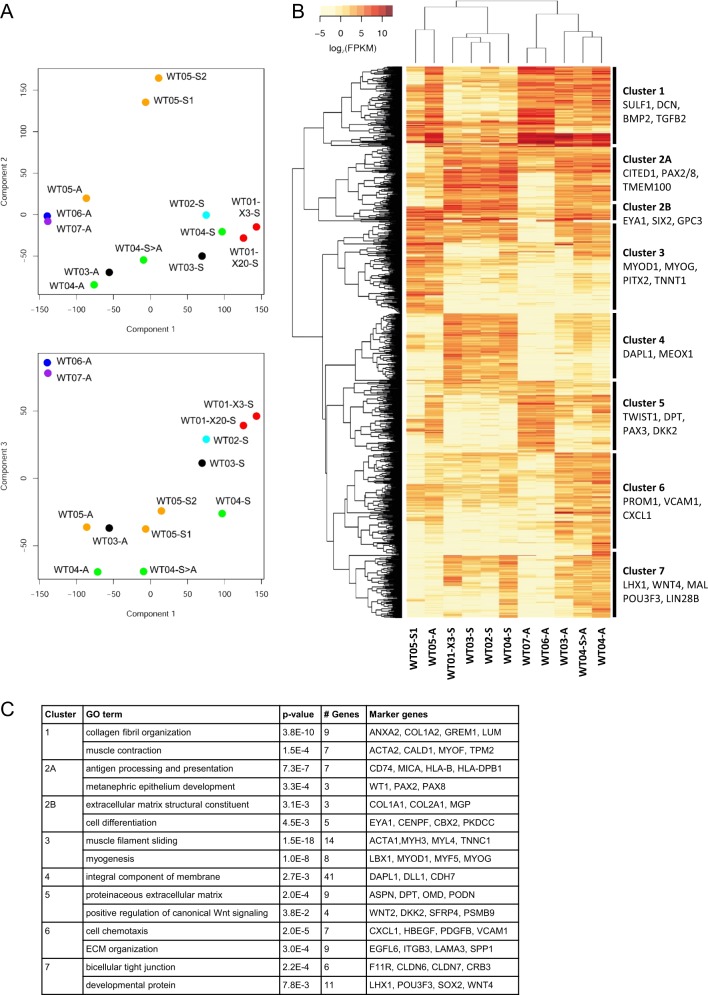
RNA expression profiling of WT cell cultures. Spheroid cultures (-S) with blastemal (WT01, WT02, and WT03), epithelial (WT04) and stromal (WT05) histology were analyzed by RNAseq together with primary adherent (-A), and spheroid derived adherent (-S>A) cells. To assess the impact of long-term in vivo cultivation WT01-X20-S (from the 20th xenograft passage) was included in addition to WT01-X3-S (from 3rd xenograft passage). Spheroid culture WT05-S2 (grown for 21 months) was included to study long-term in vitro changes. WT06-A and WT07-A are mesenchymal/stromal adherent cultures established with a slightly different protocol (lacking Y-27632) [18]. **a** Components PC1 (contribution 38.2%), PC2 (22.55%), and PC3 (11.37%) were used for principle component analysis of RNA expression. **b** Unsupervised clustering of 1000 most differentially expressed genes with fpkm ≥ 5 (table S2). **c** GO-term enrichment analysis of genes defining clusters 1−7

Principle component analysis (PCA) showed close clustering of blastemal and epithelial spheroid cultures, while stromal spheroids were grouped distant from all other 3D cultures (Fig. 2a). Corresponding primary adherent cell cultures (WT03-A, WT04-A, and WT05-A) clustered closer to previously established adherent cultures WT06-A and WT07-A, while spheroid derived adherent cells (WT04-S>A) were classified in between corresponding spheroid and adherent cultures. Blastemal spheroids derived from xenograft passages 3 and 20 were closely related as well as stromal spheroids differing in duration of in vitro cultivation (WT05-S1/2).

Unsupervised clustering based on 1000 most differentially expressed genes (fpkm ≥ 5 in at least one sample) with exclusion of long-term cultures (WT01-X20-S and WT05-S2) separated 3D from 2D cultures (Fig. 2b, Supplementary Table S2). The stromal spheroid culture WT05-S1/S2, together with its adherent counterpart WT05-A, was classified distant to other 3D cultures, however, and it clustered with adherent cells if genes with fpkm ≥ 1 were used (data not shown).

GO-term analysis was used together with classification markers from single cell RNAseq of murine and human kidney development [22–24] to categorize gene expression clusters (Fig. 2b, c). All spheroid cultures expressed a small set of genes involved in early kidney development (*EYA1, SIX2,* and *GPC3*), independent of spheroid histology (cluster 2B). While blastemal and epithelial spheroids showed additional high expression of genes typical for condensing mesenchyme and (primed) NPCs (*CITED1, PAX2/8, TMEM100, DAPL1*; clusters 2A and 4), stromal spheroid and adherent cultures exhibited high levels of muscle related genes (*MYOD1, MYOG, PITX2*; cluster 3). Expression of genes involved in epithelial differentiation like *WNT4* and *LHX1* was seen predominantly in epithelial spheroid and adherent cultures and to a lesser extent in blastemal spheroids (cluster 7). While stemness-related genes *LGR5*, *LIN28B*, and *POU3F3* were expressed in WT04 and WT05 derived cells, they were not detectable in blastemal spheroids.

Strong expression of ECM related genes that are typically seen in renal stromal elements (*SULF1, DCN,* and *DPT*) and of genes involved in epithelial to mesenchymal transition (*BMP2, DAB2, TGFB,* and *TWIST1*) was seen in adherent cell cultures (clusters 1, 5, and 6).

Specific comparison of 3D and corresponding 2D WT cell cultures (WT01-X3-S, WT02-S, WT03-S, WT04-S, and WT05-S vs. WT03-A, WT04-A, and WT05-A) revealed 871 genes to be differentially expressed (*q*-value < 0.05 and FC > 2; Supplementary Table S3). Of these 355 were upregulated in spheroids. Functional annotation clustering using DAVID (david.ncifcrf.gov) showed enrichment of genes involved in transcription activation, translational initiation, anterior/posterior patterning, and negative WNT-signaling. In adherent WT cells 516 genes were upregulated, related to ECM and ECM-remodeling, cell–cell and cell-matrix adhesion, as well as stress fiber formation (GO-terms and associated genes are given in Table S3).

### Plasticity of spheroids

Spheroids could be dissociated into single cell suspensions and they formed again when seeded in 96-well ultralow attachment plates. Seeding of 100 (WT04) to 10,000 (WT01) cells per well was necessary to obtain viable and proliferating spheroids.

**Fig. 3:**
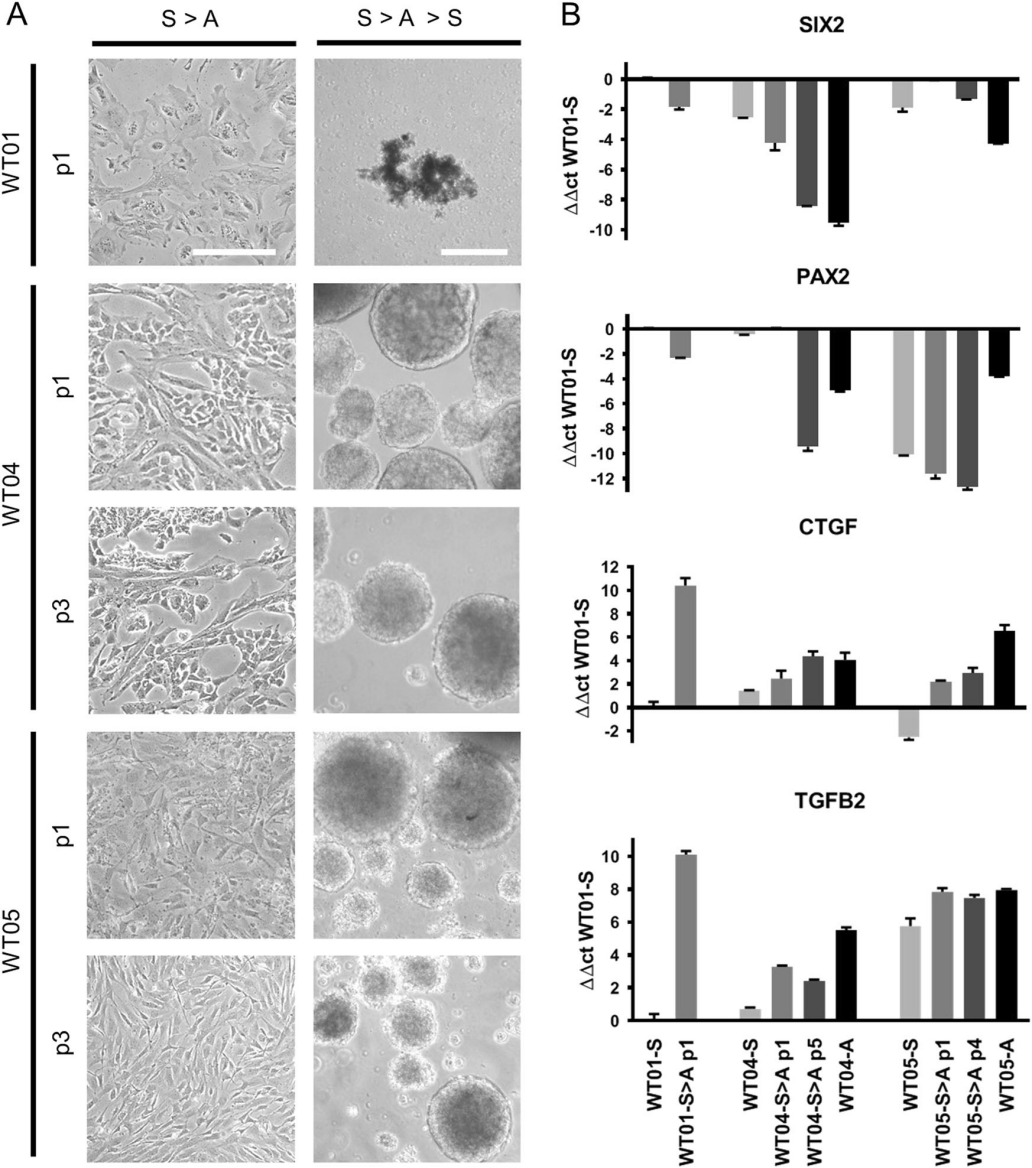
Spheroid plasticity upon adherent cultivation. **a** Spheroids dissociated into single cell suspensions could be grown adherently on cell culture grade plates (S>A). While blastemal cells quickly lose proliferation capacity (WT01 shown as an example), epithelial, and stromal derived cells could be passaged more than ten times and retained spheroid forming capacity for three passages (S>A>S). p passage. Scale bar: 200 µm. **b** Quantitative RT-PCR of *SIX2*, *PAX2*, *TGFB2*, and *CTGF* in spheroid (S), spheroid derived adherent (S>A), and corresponding primary adherent (A) cultures. For WT04 and WT05 early (S>A_p1) and later passages (S>A_p4/p5), not capable of forming spheroids, were used. Blastemal spheroid culture WT01-S was taken as a reference to calculate ΔΔct-values

All blastemal cells grew long-term under 3D conditions, but they lost proliferation capacity quickly when plated on cell culture grade plastic surfaces, where they became senescent. In contrast, cells from epithelial and stromal spheroids proliferated for more than ten passages. They even retained spheroid forming capacity when passaged three times under adherent conditions, and newly formed spheroids showed morphological features similar to the initial spheroid cultures (Fig. 3a).

To study the effects of transient adherent growth, selected genes with differential expression in spheroid vs. adherent cultures were tested by quantitative RT-PCR in spheroid, spheroid-derived, and primary adherent cultures. For blastemal WT01 cultures no corresponding primary adherent cells were available. While genes involved in early kidney development (*SIX2*, *PAX2*) were downregulated upon adherence, expression of *TGFB2* and *CTGF* that are more typical of stromal elements was induced compared with spheroid cultures (Fig. 3b). *SIX2* and *PAX2* were expressed more strongly in early vs. late spheroid derived adherent cells that had lost spheroid forming potential. There was no striking difference of *TGFB2* and *CTGF* expression in early and late passages of transient adherent cells. Thus, other differences in expression patterns may determine whether spheroids can be reformed or not and it needs to be elucidated further, which genes are required to retain spheroid forming capacity.

### Genetic manipulation of spheroid cultures

**Fig. 4:**
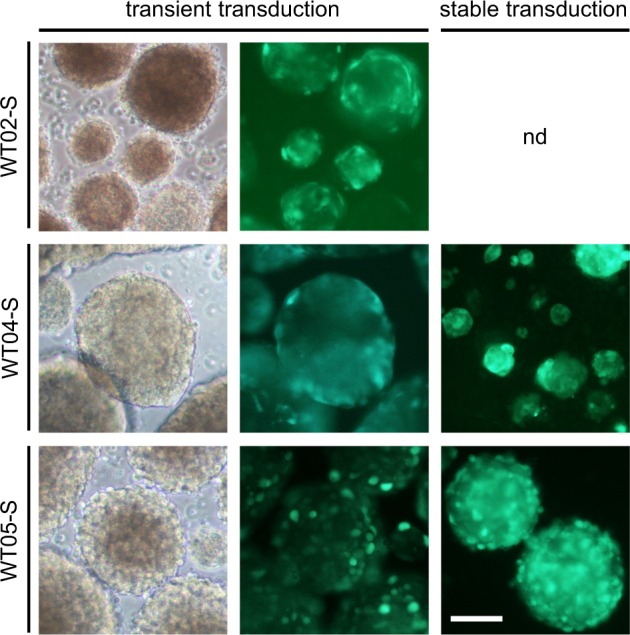
Transduction of spheroid cultures with GFP expressing vectors. pLL3.7 was used for transient transduction. For stable transduction, pGIPZ infected spheres were selected with puromycin. Scale bar: 100 µm

To assess the potential for genetic manipulation, blastemal, epithelial, and stromal spheroids (WT02, WT04, and WT05) were transduced with lentiviral vectors expressing GFP. Transduction was possible in each case, although with differences in infection rates (Fig. 4). Selection with puromycin was possible for pGIPZ infected cells and homogenous transgenic spheroids with rather uniform GFP expression could be established. Stable transduction was not successful for blastemal WT02, most likely due to insufficient multiplicity of infection reached. Since 10,000 viable cells per spheroid were necessary to maintain proliferation in blastemal cultures, optimization of the infection and selection protocol may be needed in that case.

### Xenotransplantation of spheroids

As a faithful tumor model, spheroids should retain tumor forming capacity in vivo. For WT01, primary patient tumor material had been transplanted subcutaneously in immunodeficient NSG mice, giving rise to a tumor mass of about 1 ml after 3 months. The xenograft tumor consisted of blastemal cells only and it could be serially transplanted 20 times without obvious histological changes. All xenografts possessed high sphere forming capacity in vitro and RNA sequencing showed very similar expression patterns in spheroids derived from the third (WT01-X3) and 20th (WT01-X20) xenograft passage (Fig. 2a).

**Fig. 5:**
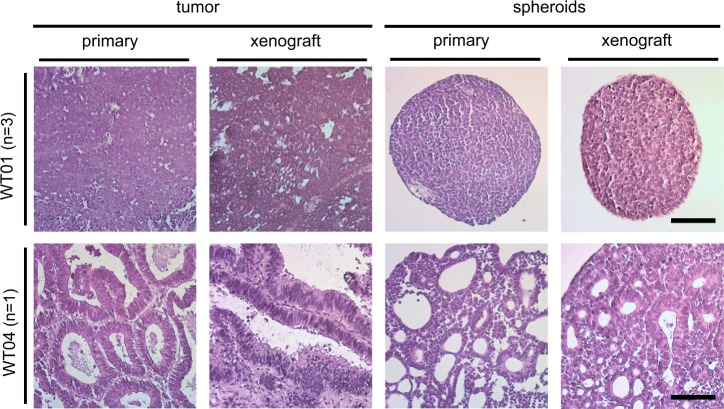
Xenograft formation. Histological comparison of primary tumors, spheroid derived xenografts, as well as primary and xenograft derived spheroids for WT01 and WT04. Xenograft transplantation was done in NSG mice (WT01: *n* = 3, WT04: *n* = 1) and tumor incidence was 100%. The experiment was terminated, when tumors reached a size of 0.8–1 ml. Scale bar: 50 µm

Importantly, spheroid cultures WT01-X3-S and WT04-S again gave rise to xenograft tumors after 3–4 months when injected subcutaneously into NSG mice. The blastemal spheroids generated a blastemal xenograft with the same histological characteristics as the initial xenograft tumor (Fig. 5). The epithelial WT04-S cells produced a tumor with epithelial and stromal elements as seen in the original tumor. Both spheroid-derived xenografts again yielded spheroid cultures in vitro that were very similar to the original cultures.

## Discussion

In vitro models represent an easy to handle and cost-effective tool to investigate tumor biology and to evaluate new treatment strategies well before clinical testing. Unfortunately, studying in particular the high-risk blastemal WT subtype has been hampered by the lack of any WT blastemal cell cultures. One reason for the failure of blastemal primary cell cultivation may be the need for specific, adapted culture conditions to keep the undifferentiated status. Here we show, that propagation of blastemal WT cells is possible, if cells are grown as 3D spheroids and in media containing ROCK inhibitor (Y-27632) to avoid anoikis. Under these conditions we could generate blastemal WT spheroids, and we were able to establish 3D cultures representing epithelial and immature stromal WT elements as well.

Even under long-term cultivation blastemal spheroids retain expression of genes typically seen in condensing mesenchyme and nephron progenitors (e.g., *SIX2*, *SALL1*, *EYA1*, *CITED1*, or *PAX2*), suggesting that blastemal cells are trapped in early renal development and retain this undifferentiated status. The expression pattern was very similar in epithelial spheroids, while stromal spheroids still expressed high amounts of SIX2, but none of the other markers. Shukrun et al. [21] described a critical, strong expression of ALDH1 in tumor initiating cells from blastemal xenografts, visualized by metabolic labeling, and FACS analysis. This was not seen in our experiments based on RNAseq data, although this approach may bias against a rare stem cell population. Only one blastemal and the epithelial culture showed expression of *ALDH1A2*, the only *ALDH1* gene expressed, at an appreciable level (not shown). Furthermore, none of the spheroid cultures expressed stemness related genes like *SHH*, *OCT4*, *KLF4,* or *LIN28A*, that were enriched in WT cancer stem cells in the analysis by Shukrun et al. Only stromal and in part epithelial spheroids showed moderate expression of the stemness-related genes *LGR5*, *LIN28B*, and *POU3F3*. The absence of these genes in blastemal spheroids suggests alternative pathways that ensure self-renewing capacity and proliferation in vitro, while stromal spheres may be more dependent on classical stemness genes.

Since blastemal tumor cells likely originate from undifferentiated renal precursor cells, in vitro studies on NPCs may serve as a reference for our spheroid protocol. Li et al. [25] described propagation of murine and human NPCs as floating 3D aggregates that maintain nephrogenic potential during long-term expansion. These NPC spheres are morphologically similar to our blastemal WT spheroids and they also did not survive as single cells but needed about 10,000 cells to form a proliferating sphere. In contrast to wild-type NPCs, WT spheroids do not rely on elaborate growth factor cocktails, however. A recent protocol to establish kidney “microorganoids” likewise included suspension culture steps [26] and these microorganoids appear quite similar to our epithelial WT spheroids, with visible tubular structures on brightfield images and histological sections. The fact that blastemal WT cells as well as NPCs can be propagated efficiently under suspension conditions suggests that spheroid cultures are better suited to maintain the undifferentiated state of these cells.

Our spheroid protocol appears to work best for blastemal WT cells. Three out of five successful cultures contained mostly blastema and additional cultures that we have established since (data not shown) likewise showed blastemal morphology, indicative of a selective advantage. Given the phenotypic and probably ontogenetic diversity of WT it may be difficult or even impossible to find universal culture conditions that support proliferation of all WT cell types to the same extent. On the other hand, stromal cells can be cultivated effectively under adherent conditions and they retain proliferation capacity in many cases [18]. Since we selected for nonadherent primary cells in our current protocol, we may specifically reduce the number of stromal cells in 3D cell aggregates.

Despite several attempts, epithelial spheroids could only be established from a single tumor harboring a *TP53* mutation. This mutation might induce additional alterations that facilitate primary cell cultivation in this case. Even though epithelial spheres showed tubular structures, there is a morphological continuum to less differentiated blastemal spheres that may correspond to early steps of epithelial differentiation. It is conceivable that cultivation of epithelial WT cells is more efficient with different media compositions and an alternative 3D “organoid” approach. While organoid technology was developed for epithelial stem cells, it has been applied successfully to different epithelia-derived types of cancer as well [27]. Epithelial cells from human adult kidneys can be grown as “tubuloids” using an organoid approach [28]. In a first test with WT, these samples could be grown as “tumoroids” with primarily epithelial appearance. It remains to be seen if admixtures of all three WT elements can be propagated long-term under organoid conditions, or whether the organoid strategy preferentially promotes cultivation of epithelial cells as seen with other malignancies.

An area yet to be explored is the epigenetic status of WT cultures. Epigenetic changes especially at chromosome 11p15 are frequent in WT [5, 7, 29]. Our cultures offer new possibilities to modulate epigenetic marks in vitro and to unravel their contribution to the malignant state.

The simple and low-cost method described here establishes long-term spheroid cultures, which maintain characteristics especially of blastemal tumor elements and represent an in vitro model for this high-risk WT subtype. Spheroid cultures proved to be stable upon long-term cultivation with respect to both gene expression and phenotype and they maintained features of the initial tumor material. The cultures are amenable to genetic manipulation by viral transduction and thus allow functional studies of candidate genes. The ease of generating larger numbers of uniform spheres in multiwell format will facilitate high-throughput screening. In addition, multiple xenograft tumors can be generated in parallel from spheroid cultures to extend drug testing to the in vivo situation.

## Methods

### Patients and sample preparation

Tumor material and control tissue or blood was obtained from the SIOP2001/GPOH WT study. Clinical data and reference pathology were available from the clinical study registry. Genomic DNA and RNA of primary cells, tumor and control tissue were isolated as described before [18]. Written consent for tumor banking and research use was obtained according to national regulations including ethical approval (Ethikkommission der Ärztekammer des Saarlandes. Germany; No.: 136/01 and 248/13).

### Cell culture

Primary WT cultures were started within 24 h after tumor nephrectomy. All cells were cultivated in D10Y medium (DMEM high glucose, 10% fetal calf serum, 1% penicillin/streptomycin, 10 µM ROCK inhibitor Y-27632 (Selleckchem)) at 37 °C with 5% CO_2_.

Viable tumor tissue was minced with scalpels and treated with 250 U/ml collagenase I (Merck) and 1 mg/ml DNase 1 (Roche) for 30–60 min at 37 °C with intermittent pipetting, without aiming for a single cell suspension. After centrifugation cells were plated on cell culture dishes in D10Y for 5–12 h, to allow for adherence of cells that are not able to form floating aggregates. Adherent cells were cultivated as described before [18].

Supernatant containing cell aggregates (spheroids) was transferred to bacterial petri dishes and kept under continuous shaking at 50 rpm (Celltron, Infors HT). For small culture volumes or if cells formed large clumps upon shaking, ultralow attachment plates (Corning) were used without shaking. Medium was changed every 2–3 days. Once spheroids reached a diameter of 1 mm, they were mechanically disrupted using 200 µl plastic pipette tips. For cryopreservation, spheroid cultures were gently dissociated, resuspended in freezing medium (90% FCS, 10% DMSO), and stored in liquid nitrogen.

For spheroid derived adherent cells (S>A), spheres were mechanically dissociated and the cell suspension was plated on cell cultures dishes in D10Y. Once cells attached, they were passaged like adherent cell cultures.

For comparison two adherent stromal WT cultures (WT06, WT07) generated by a prior protocol and subjected to a more limited characterization were included as a reference for stromal cells [18].

### Mouse xenografts

Xenografts were established in NSG mice (NOD.Cg-*Prkdc*^*scid*^ *Il2rg*^*tm1Wjl*^/SzJ, Jackson Laboratory), starting from primary human tumor material or spheroid cultures. Tumor material was minced mechanically and about five tumor pieces of 1 mm^3^ were used for subcutaneous transplantation into the hind flank. Spheroids were injected subcutaneously with a 21 G needle, using 5–10 spheroids in 50 µl PBS. Mice were monitored daily for up to 120 days. The experiments were terminated when tumors reached 1 cm diameter. Xenograft material was partitioned to allow for retransplantation, primary cell culture, formalin fixation for histological analyses, and cryopreservation for DNA and RNA isolation. All experiments involving mice were authorized by the local ethics committee (government of Lower Franconia, Germany, project license numbers 01-11 and 99-12) and carried out in accordance with institutional and European Union guidelines for animals in scientific research.

### Immunohistochemical (IHC) staining

H&E staining and IHC analyses were performed on 5 µm sections of FFPE (formalin-fixed paraffin embedded) tumor tissue and spheres. Staining was performed according to standard protocols using HiDef Detection™ HRP Polymer System (Medac, Germany) and DAB detection. Primary antibodies used are listed in supplementary methods.

### MLPA and LOH analysis

MLPA analysis (multiplex ligation-dependent probe amplification, SALSA-MLPA-P380, MRC Holland) to determine copy number alterations was conducted as described [30]. Allelic status (LOH, loss of heterozygosity) was analyzed by PCR amplification of microsatellite markers using primers listed in supplementary methods.

### Whole exome sequencing

WES was done by Novogene (UK) using the Agilent SureSelect Human All Exon V6 kit (Agilent Technologies, USA) with paired end sequencing (PE-150) to yield 40 million reads on average. Reads were mapped to the human reference genome (hg19) and analyzed as described in supplementary methods. Briefly, SNVs, indels, and CNVs were identified and variants that affect protein sequence or splice sites but are rare in the population were characterized further. Detailed information can be found in supplementary material.

### RNA sequencing

Transcriptome sequencing was done by BGI Tech (HongKong) on a BGIseq500 platform (hexamer-primed oligo-dT selected RNA, 100 bp paired end, ~35 million reads). Details can be found in supplement. After mapping and annotation, PCA and differential expression were calculated. In addition, SNVs and indels were identified and evaluated for putative driver events.

### Realtime RT-PCR

cDNA was prepared using the RevertAid first strand cDNA synthesis kit with oligo-dT primers (ThermoFisher). SybrGreen based quantification was done on a Realplex cycler (Eppendorf) as described before [31]. Primers are listed in supplementary methods. All measurements were performed in duplicates and mean values were calculated. *HPRT* was used to normalize expression levels.

### Viral transduction

GFP-expressing lentiviral constructs pLL3.7 (Addgene) and pGIPZ (Open Biosystems) were used for transient and stable transduction of spheroids. Viral supernatants were produced as described previously [18]. For infection, spheroids were gently disrupted with 200 µl plastic tips and incubated with virus containing supernatant in the presence of 8 µg/ml polybrene and 10 µM Y-27632 for 6 h in ultralow attachment plates (Corning). After 48 h pGIPZ infected spheroids were selected with 0.5 µg/ml puromycin for at least 2 weeks to obtain stable GFP-expressing spheroid cultures.

## Supplementary information

List of supplemental Information

Supplementary figure S1

Supplementary figure S2

Supplementary methods

Supplementary table S1

Supplementary table S2

Supplementary table S3
