# Supplementary material for: High-risk blastemal Wilms tumor can be modeled by 3D spheroid cultures in vitro
Source: Oncogene. 2019 Sep 27;39(4):849–61. doi: 10.1038/s41388-019-1027-8 (PMC6976522; doi:10.1038/s41388-019-1027-8)
Supplement: Supplementary file 1 — List of supplemental Information [file 41388_2019_1027_MOESM1_ESM.docx]

**Supplementary Information**

**Supplementary figure S1:** Copy number variations of primary tumor material based on WES data

**Supplementary figure S2:** H&E and IHC staining of primary tumor material used to generate spheroids.

**Supplementary table S1:** Somatic SNV of primary tumor material and corresponding spheroids

**Supplementary table S2:** Fpkm values of the 1000 most differentially expressed genes in WT cultures

**Supplementary table S3**: Differentially expressed genes in spheroids vs. adherent cultures

**Supplementary methods:**

Detailed information on whole exome and RNA sequencing, primary antibodies for IHC**,** primers used for LOH analysis and quantitative RT-PCR
