# Supplementary material for: High-risk blastemal Wilms tumor can be modeled by 3D spheroid cultures in vitro
Source: Oncogene. 2019 Sep 27;39(4):849–61. doi: 10.1038/s41388-019-1027-8 (PMC6976522; doi:10.1038/s41388-019-1027-8)
Supplement: Supplementary file 2 — Supplementary figure S1 [file 41388_2019_1027_MOESM2_ESM.pdf]

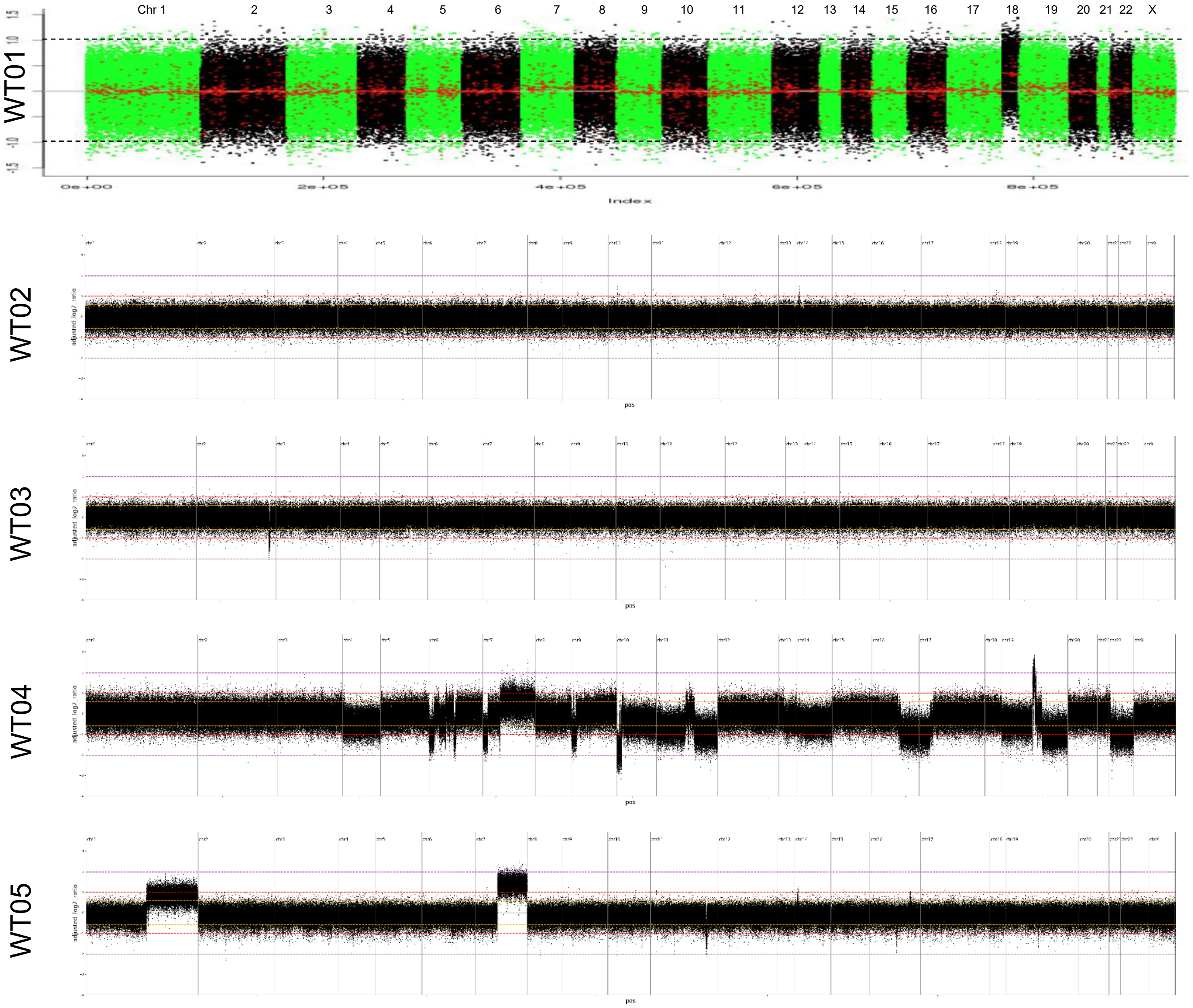

Fig. S1: Somatic CNV of primary tumor material based on WES data.

CNVs were determined by tumor to control comparison of WES reads using Varscan2. Each graph represents the adjusted log2 ratio along the genome and chromosome numbers are given above. Gains appear as values above 1, losses as values below -1. WT01 was analyzed and described previously [7].
