# Supplementary material for: High-risk blastemal Wilms tumor can be modeled by 3D spheroid cultures in vitro
Source: Oncogene. 2019 Sep 27;39(4):849–61. doi: 10.1038/s41388-019-1027-8 (PMC6976522; doi:10.1038/s41388-019-1027-8)
Supplement: Supplementary file 3 — Supplementary figure S2 [file 41388_2019_1027_MOESM3_ESM.pdf]

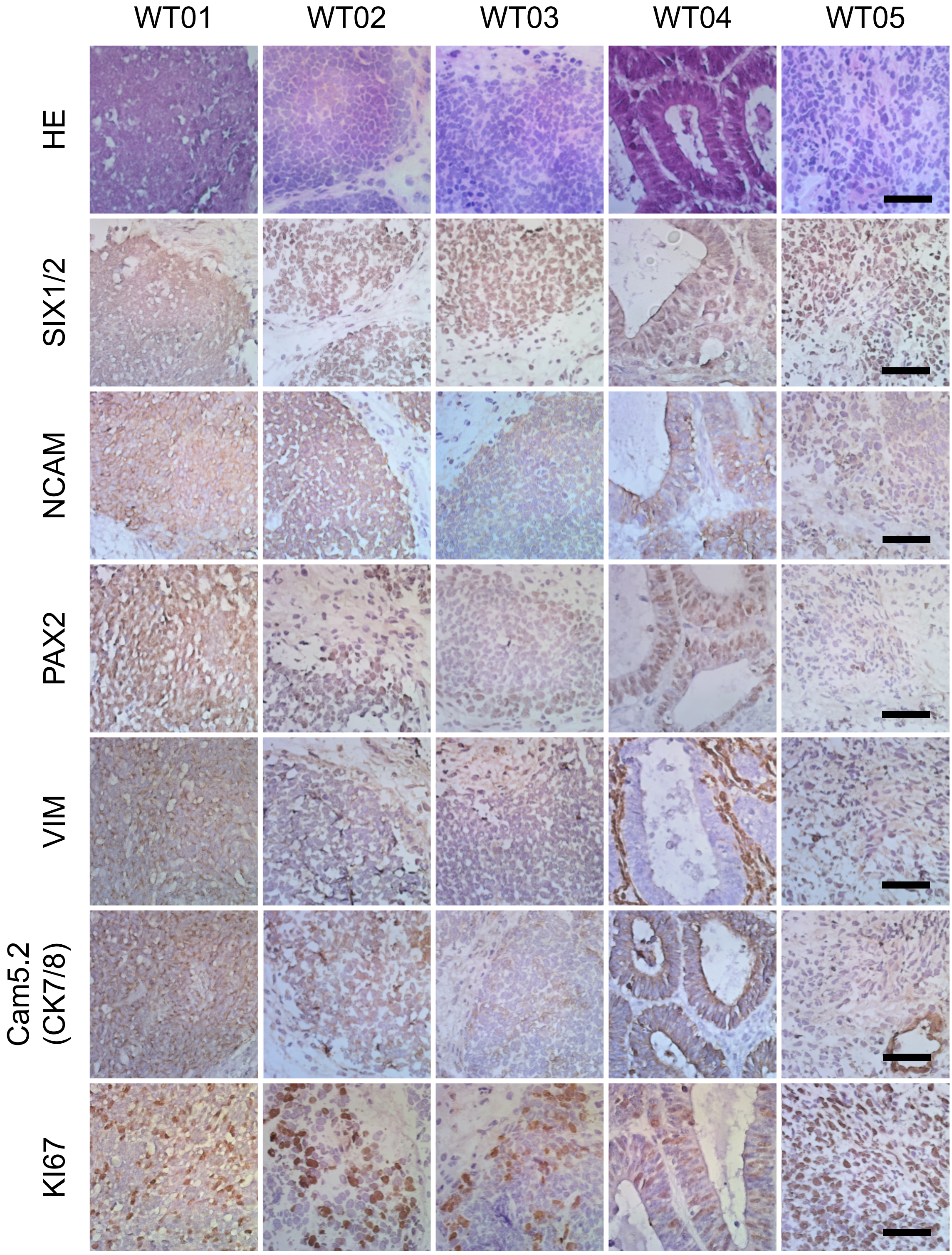

Fig. S2 H&E and IHC staining of primary WT tumor material used to generate spheroids. H&E staining of tumor material (row 1) as shown in figure 1. IHC staining for renal progenitor/blastemal markers (SIX1/2, NCAM, PAX2), the mesenchymal marker vimentin (VIM), the epithelial marker cytokeratin 7/8 (Cam5.2) and the proliferation marker KI-67, as done for spheroid material. scale bar: 25 µm
