# Supplementary material for: High-risk blastemal Wilms tumor can be modeled by 3D spheroid cultures in vitro
Source: Oncogene. 2019 Sep 27;39(4):849–61. doi: 10.1038/s41388-019-1027-8 (PMC6976522; doi:10.1038/s41388-019-1027-8)
Supplement: Supplementary file 4 — Supplementary methods [file 41388_2019_1027_MOESM4_ESM.pdf]

Supplementary methods

Whole exome sequencing (WES)

Whole exome sequencing was done by Novogene (UK) using the Agilent SureSelect Human All Exon V6 kit (Agilent Technologies, CA, USA) with paired end sequencing (PE-150) to yield 40 million reads on average. An initial quality assessment was performed using FastQC, v0.11.5 (Available online at: http://www.bioinformatics.babraham.ac.uk/projects/fastqc). Adapters and low-quality reads were trimmed with TrimGalore, v0.4.0 (http://www.bioinformatics.babraham.ac.uk/projects/trim_galore/) powered by Cutadapt, v1.8 (https://cutadapt.readthedocs.io/en/stable/).

The trimmed reads were mapped to the human reference genome (hg19) using BWA mem, v0.7.12 (Li and Durbin, 2009). Sorting and indexing were executed using Picard, v1.125 (available online at: http://broadinstitute.github.io/picard/) and SAMtools, v1.3 [1], respectively. Duplicate reads were marked using Picard. Local indel realignment was performed with GATK, v3.5 [2] which was also used for coverage calculations and germline variant calling (UnifiedGenotyper). Somatic single nucleotide variants (SNVs) were identified with MuTect1, v 1.1.4 [3] and VarScan2, v2.4.1 [4]. Small somatic insertions or deletions were detected using samtools (mpileup) with VarScan2 and Scalpel, v0.5.3 [5]. All variants were annotated with ANNOVAR, v2017-06-01 [6] and visually examined using the Integrative Genomics Viewer v2.3.68 [7]. Variants that have an impact on the protein sequence or affect a splice site are rare in the population (below a frequency of 2 % in 1000g2015aug_all, ExAC_nontcga_ALL, gnomAD_exome_ALL and gnomAD_genome_ALL). Only events where the position is covered by at least 20 reads and the alternative allele is covered by at least 8 reads and comprised at least 5% were reported. Germline variants fulfilling these criteria were reported if they were classified pathogenic or likely pathogenic in clinvar_20170905. Additionally, Varscan2 was used to detect larger copy number variations (CNVs) and loss of heterozygosity (LOH) events. CNVs were visualized using an inhouse script. LOH was reported, if more than 100 SNPs per region were affected and LOH did not occur due to copy number gain.

RNA sequencing

Transcriptome sequencing was done by BGI Tech (HongKong) on a BGIseq500 platform (hexamer-primed oligo-dT selected RNA, 100 bp paired end, approximately 35 million reads). The initial quality assessment as well as adapter and read trimming were performed as described in the whole exome sequencing section. Trimmed reads were mapped to the ENSEMBL human reference genome (ftp://ftp.ensembl.org/pub/release-75//fasta/homo_sapiens/dna/Homo_sapiens.GRCh37.75.dna.primary_assembly.fa.gz) using STAR v2.5.3a (https://github.com/alexdobin/STAR) [8]. Samtools, v1.3 [1] using htslib, v1.3 (http://www.htslib.org/) was used for sam-to-bam-conversions as well as sorting and indexing of the alignment files. For gene annotation, the ENSEMBL human annotation (ftp://ftp.ensembl.org/pub/release-75/gtf/homo_sapiens/Homo_sapiens.GRCh37.75.gtf.gz) was used.

FPKM-values and differential expression were calculated with the Cufflinks package, v2.2.1 [9] with the no-effective-length-correction and compatible-hits-norm options in effect.

PCA analysis was performed on all cells using log_2-transformed FPKM-values of all genes expressed in more than one sample (with FPKM>=5) and a coefficient of variation >= 0.5. PCA was performed with the prcomp function from the R's stats package, v3.4.4. Subsequently, 1000 genes with the highest PC loadings (highest absolute correlation coefficient with one of the first three components) were identified and unsupervised complete linkage clustering was performed on the rows and columns using the Euclidian distance metric with the heatmap.2 function in the gplots R-package, v3.0.1.

Primary antibodies for IHC

Target	AB clone	Company	Target retrieval#	Dilution
Ki67	MIB1	Dako	CA 6.0	1:800
SIX1/SIX2*	HPA001893	Sigma Aldrich	CA 6.0	1:2000
NCAM (CD56)	123C3	Dako	TR 9.0	1:100
PAX2	PCRP-PAX2-1A7-s	Developmental Studies Hybridoma Bank	CA 6.0	1:100
VIM	AMF-17b	Developmental Studies Hybridoma Bank	CA 6.0	1:1000
pan-Cytokeratin	Cam5.2	BD Biosciences	CA 6.0	1:6

* Specified as SIX1 antibody, but detects SIX1 and SIX2 in Western blot and IF analyses.

CA 6.0: Citric acid 20 mM pH 6.0; TR 9.0: TR pH 9.0 (Dako, Hamburg, Germany)

Primers used for LOH analysis and quantitative RT-PCR

LOH analysis

Chr. region	Primer	Sequence
1p34	D1S3721-5<br>D1S3721-3	CTCCATAATCATGTGAGCAAAA<br>GTTCTCCAGAGAACGGAACC
1q23	D1S1679-5<br>D1S1679-3	CAGCCATCAAGAAAACTAGTACTGC<br>CCATGGTACTCAGCAGTGCTTTA
11p15	TH01-5<br>TH01-3	CATTGGCCTGTTCCTCCCTT<br>CACAGGGAACACAGACTCCA
11p15	D11S1392-5<br>D11S1392-3	ATCCATCTCATCCCATCATT<br>TCAACAAGACAGTACTGGCG
11q21	D11S934-5<br>D11S934-3	GCTGTCCCTGACAACTACATGC<br>TTCCATCAGAACTGGGAATGAG
11q23	D11S1987-5<br>D11S1987-5	CAGTCGCGTCTGCTTC<br>CAGACAGAGGGAAACACTGTACAT
16q12	D163253-5<br>D163253-3	TGTTCAAGCATGGGTGGA<br>CTCGAGTATCCTTCTAGAAAGTTCTATCTAT
16q12	D16S540-5<br>D16S540-3	CCTCCATAATCATGTGAGCC<br>GTGTCTTAATCTCCCCAACTCAAGACC
16q12	D16S3112-5<br>D16S3112-3	TACTTTGGAGCCCGAGG<br>AGCCCCCAGTGGTGTTTAT
16q24	D16S539-5<br>D16S539-3	CCTCTTCCCTAGATCAATACAGAC<br>CTCTGTTTTGTCTTTCAATGATATC
22q13	D22417-5<br>D22417-3	GTATGACACGGCAAGACCC<br>TTTCTTCCCTAATTGATTTCTTTT

quantitative RT-PCR

Gene	Primer	Sequence
CTGF	hCTGF-q1	GAAGAGAACATTAAGAAGGGCAAA
	hCTGF-q2	ATGTCTTCATGCTGGTGCAG
HPRT	hHPRT-q1	TGACACTGGCAAAACAATGCA
	hHPRT-q2	GGTCCTTTTCACCAGCAAGCT
PAX2	hPAX2-q1	AGGCATCAGAGCACATCAAATCAG
	hPAX2-q2	TCAGGGTTGGTGGATGCAGATA
SIX2	hSIX2-q1	CACAGGTCAGCAACTGGTTCA
	hSIX2-q2	CAGCGGGTTGTGGCTGTTA
TGFB2	hTGFb2-q1	GCATGCCCGTATTTATGGAG
	hTGFb2-q2	TTGGGTGTTTTGCCAATGTA

References:

1 Li H, Handsaker B, Wysoker A, Fennell T, Ruan J, Homer N *et al*. The Sequence Alignment/Map format and SAMtools. *Bioinformatics* 2009; 25: 2078-2079.

2 McKenna A, Hanna M, Banks E, Sivachenko A, Cibulskis K, Kernytsky A *et al*. The Genome Analysis Toolkit: a MapReduce framework for analyzing next-generation DNA sequencing data. *Genome Res* 2010; 20: 1297-1303.

3 Cibulskis K, Lawrence MS, Carter SL, Sivachenko A, Jaffe D, Sougnez C *et al*. Sensitive detection of somatic point mutations in impure and heterogeneous cancer samples. *Nat Biotechnol* 2013; 31: 213-219.

4 Koboldt DC, Zhang Q, Larson DE, Shen D, McLellan MD, Lin L *et al*. VarScan 2: somatic mutation and copy number alteration discovery in cancer by exome sequencing. *Genome Res* 2012; 22: 568-576.

5 Fang H, Bergmann EA, Arora K, Vacic V, Zody MC, Iossifov I *et al*. Indel variant analysis of short-read sequencing data with Scalpel. *Nat Protoc* 2016; 11: 2529-2548.

6 Wang K, Li M, Hakonarson H. ANNOVAR: functional annotation of genetic variants from high-throughput sequencing data. *Nucleic Acids Res* 2010; 38: e164.

7 Robinson JT, Thorvaldsdottir H, Winckler W, Guttman M, Lander ES, Getz G *et al*. Integrative genomics viewer. *Nat Biotechnol* 2011; 29: 24-26.

8 Dobin A, Davis CA, Schlesinger F, Drenkow J, Zaleski C, Jha S *et al*. STAR: ultrafast universal RNA-seq aligner. *Bioinformatics* 2013; 29: 15-21.

9 Trapnell C, Williams BA, Pertea G, Mortazavi A, Kwan G, van Baren MJ *et al*. Transcript assembly and quantification by RNA-Seq reveals unannotated transcripts and isoform switching during cell differentiation. *Nat Biotechnol* 2010; 28: 511-515.
